# Development of a web application to evaluate spirometric curve and clinical variables to support COPD diagnosis in primary care

**DOI:** 10.7705/biomedica.7142

**Published:** 2024-05-31

**Authors:** Adriana Maldonado-Franco, Luis F. Giraldo-Cadavid, Eduardo Tuta-Quintero, Alirio R. Bastidas, Angélica Moreno-Giraldo, Daniel A. Botero-Rosas

**Affiliations:** 1 Facultad de Ingeniería, Universidad de La Sabana, Chía, Colombia Universidad de la Sabana Facultad de Ingeniería Universidad de La Sabana Chía Colombia; 2 Facultad de Medicina, Universidad de La Sabana, Chía, Colombia Universidad de la Sabana Facultad de Medicina Universidad de La Sabana Chía Colombia; 3 Servicio de Neumología Intervencionista, Fundación Neumológica Colombiana, Bogotá, D. C., Colombia Servicio de Neumología Intervencionista Fundación Neumológica Colombiana Bogotá, D. C. Colombia

**Keywords:** Pulmonary disease, chronic obstructive, diagnosis, spirometry, data accuracy, enfermedad pulmonar obstructiva crónica, diagnóstico, espirometría, exactitud de los datos

## Abstract

**Introduction.:**

Choric obstructive pulmonary disease (COPD) is the third mortality cause in the world, and the development of useful diagnostic tools is necessary to improve timely diagnostic rates in primary care settings.

**Objective.:**

To develop a web application displaying spirometric and clinical information - including respiratory symptoms and risk factors- to facilitate a COPD diagnosis.

**Materials and methods.:**

In this cross-sectional study, an expert consensus was carried out with three specialists using the Delphi method to choose the relevant variables for COPD diagnosis. We developed a Python-based web application to diagnose COPD, displaying the clinical variables deemed relevant by the experts along the spirometric curve.

**Results.:**

Twenty-six clinical variables were included in the web application for the diagnosis of COPD. A fourth expert used the web application to classify a cohort of 695 patients who had undergone spirometry in a third-level centre and had answered at least one of five questionnaires for COPD screening. Out of the 695 subjects, 34% had COPD, according to the expert that diagnosed them using the web application. Only 42% of the patients in the COPD group had received a previous COPD diagnosis and 19% of the patients in the no COPD group had been misdiagnosed with the disease.

**Conclusion.:**

We developed a web application that displays demographic and clinical information, as well as spirometric data, to facilitate the process of diagnosing COPD in primary care settings.

## Resumen

**Introducción.:**

La enfermedad pulmonar obstructiva crónica (EPOC) es la tercera causa de mortalidad en el mundo y es necesario el desarrollo de herramientas diagnósticas útiles para mejorar las tasas de diagnóstico oportuno en los entornos de atención primaria.

**Objetivo.:**

Desarrollar una aplicación web que muestre la información clínica y de la espirometría -incluyendo síntomas respiratorios y factores de riesgo- para facilitar el diagnóstico de la EPOC.

**Materiales y métodos.:**

En este estudio transversal se realizó un consenso de expertos con tres especialistas usando el método Delphi para elegir las variables relevantes para el diagnóstico de EPOC. Se desarrolló una aplicación web basada en Python que muestra la información clínica relevante según los expertos, junto con la curva y los datos de la espirometría para el diagnóstico de la EPOC.

**Resultados.:**

Se incluyeron 26 variables clínicas para el diagnóstico de la EPOC. Un cuarto experto utilizó la aplicación web para clasificar una cohorte de 695 pacientes a los que se les había realizado una espirometría en un centro de tercer nivel y que habían contestado al menos uno de los cinco cuestionarios para la detección de la EPOC. De los 695 sujetos, el 34 % tenían EPOC según el experto que les diagnosticó usando la aplicación web. Sólo el 42 % de los pacientes del grupo con EPOC había recibido un diagnóstico previo de la enfermedad y el 19 % de los pacientes del grupo sin EPOC había sido diagnosticado erróneamente con la enfermedad.

**Conclusión.:**

Se desarrolló una aplicación web que muestra información demográfica y clínica, así como datos espirométricos, para facilitar el proceso de diagnóstico de la EPOC en entornos de atención primaria.

According to the World Health Organization (WHO), chronic obstructive pulmonary disease (COPD) is a public health issue because it is the third mortality cause worldwide ^1^. COPD is characterised by progressive airflow limitation that leads to persistent respiratory symptoms, and it is usually caused by exposure to smoke and other harmful gases or particles ^2^. Its timely diagnosis is critical to improve the patient’s quality of life by guaranteeing appropriate management of symptoms and stimulating protective measures such as smoke cessation ^3^.

The current gold standard for diagnosing COPD is measuring in the spirometry curve the ratio between the forced expiratory volume in the first second (FEV_1_) and the forced vital capacity (FVC) after applying a bronchodilator. According to the Global Initiative for Chronic Obstructive Lung Disease (GOLD), if a patient has one or more risk factors, chronic respiratory symptoms, and ( spirometric FEV_1_/FVC ratio is below 0.7, the subject is deemed to have COPD ^2^.

As an alternative, the American Thoracic Society (ATS) and the European Respiratory Society (ERS) advise using the statistically derived lower limit of normal value as a threshold for diagnosis, which better displays the normal effect of ageing in pulmonary function ^4^^,^^5^. However, this approach is known to have a large misdiagnosis rate.

The PLATINO study ^6^, which included patients in five cities in different Latin-American countries, found that 88.7% of the COPD cases had not been previously diagnosed. Meanwhile, 63.7% of the patients that had a previous COPD diagnosis did not fulfil the GOLD criteria of a post-bronchodilator FEV_1_/FVC ratio < 0.7. More recently, the PUMA study ^7^ found an overall underdiagnosis (77%) and misdiagnosis (30.4%) rates in Argentina, Colombia, Uruguay, and Venezuela.

Despite knowing that the FEV_1_/FVC ratio is an imperfect gold standard, most research that proposes new metrics and methods for diagnosing COPD uses this ratio as reference ^8^^-^^11^. However, the diagnostic process should include clinical variables and the shape of the spirometric curve to satisfy international guidelines. Therefore, we created a new web application that displays relevant information obtained during regular clinical practice to support decision-making of a medical professional when diagnosing COPD.

This study identifies the essential demographic and clinical data to improve the diagnosis of COPD through a structured expert consultation, and it presents the development of a web application integrating demographic, clinical and spirometry data relevant to online patient assessment.

Additionally, it describes the classification of a cohort of patients performed by an expert using the developed web application.

## Materials and methods

We conducted a cross-sectional study approved by the Ethics Committee of the *Clinica Universidad de La Sabana*, a tertiary level centre. We had access to a database of 765 patients who underwent spirometry between August 2017 and August 2019, and all patients had authorized the use of their data for research by signing an informed consent form.

### Questionnaires

The patients had completed at least one of the five questionnaires designed for COPD detection as part of their clinical diagnostic process. Including this information in the web application would allow us to provide a more comprehensive and detailed insight into the evaluated patients. Each questionnaire comprises a set of inquiries related to age, respiratory symptoms, and risk factors. They vary in their maximum scores and thresholds for identifying COPD risk.

The Chronic Obstructive Pulmonary Disease-Population Screening (COPD-PS) questionnaire consists of five multiple-choice questions, each scored from 0 to 2, with a maximum score of 10 and a threshold of 4 ^12^^,^^13^.

The Lung Function Questionnaire (LFQ) features five Likert-scale questions, scored from 1 to 5, with a maximum score of 25 and a threshold of 18 ^14^^,^^15^.

The COPD Diagnostic Questionnaire (CDQ) contains eight questions, with a threshold of 19 ^16^^,^^17^.

The PUMA Questionnaire comprises seven questions, some scored from 0 to 1 and others from 0 to 2, yielding a maximum score of 9 and a threshold of 5 ^7^^,^^18^.

Lastly, the “Could it be COPD?” questionnaire presents five yes/no questions, scored as 1 for yes and 0 for no, with a maximum score of 5 points and a threshold of 3 ^19^.

### Spirometry

Raw spirometry records of the patients were stored in fvl format in a CareFusion Vmax Encore 22 PFT Machine (CareFusion, Yorba Linda, California) at the centre’s Pulmonary Function Laboratory. Each file had the patient’s ID, full name, test date and the record for time, volume, and flow. The reference values for the predicted and lower limit of normal of FVC and FEV_1_ were calculated based on the equations obtained in the National Health and Nutrition Examination Survey (NHANES III) ^20^.

### Delphi method

The general areas covered by the COPD questionnaires were submitted for expert consideration through a series of questionnaires designed in Google Forms, according to the Delphi method ^21^. Delphi method is a consensus research technique used when available information is insufficient to perform a precise analysis, and collective specialist judgment is considered valuable.

Participants had to be experts (appropriate education, background, and work experience) in their field, and the number of participants should be sufficient and available for results verification through follow-up survey research.

The method consisted of rounds or iterations of questions asked to the participants; the researchers received and summarised the results and then provided that information back to the participants for them to reconsider their previous responses. After three rounds of questioning over two months, a consensus was reached. This technique facilitated reaching a consensus among an expert panel when money and time do not allow for frequent meetings ^22^^,^^23^.

For this project, we invited three pneumologists to participate. These experts also specialised in internal medicine, received frequently patients with obstructive conditions, and are interested in studying COPD. The individuals who participated in the Delphi method were not part of the study.

### Web application

We developed the web application in Python 3.0. It includes variables that were deemed as relevant by the experts, along with the best spirometry- obtained post-bronchodilator flow-volume curve, and a table that displayed the lower limit of normal and the predicted values for FEV_1_ and FVC and the best of these values measured by spirometry before and after applying a bronchodilator.

A fourth expert (specialist in internal medicine, pneumology and epidemiology) evaluated each patient record using only the information in the web application. He was able to label each subject as diseased with COPD, healthy, and he also had the chance of labelling the case as “Non-conclusive” with the corresponding input reason. The patients were classified in two groups: “COPD2 or “No COPD” according to the expert. This classification was used as reference test to evaluate the spirometric measurements performance.

### Inclusion/exclusion criteria

The inclusion criteria were any patient included in the repository available in the spirometer who had answered at least one of the questionnaires, with a signed Informed consent form and under study due to suspicion of respiratory disease. The exclusion criteria were records of patients under 40 years old, repeated or misnamed patient records, patients without height measurement (necessary to calculate the lower limit of normal and predicted values for FVC and FEV_1_), patients with incomplete information regarding the variables deemed relevant by the expert consensus, spirometry with less than three post-bronchodilator trials, and spirometric record that could not be interpreted by the expert due to bad acquisition technique or noise.

### Statistical analysis

Categorical variables were described in terms of absolute and relative frequencies. The distribution of quantitative variables was assessed by the one-sample Kolmogorov-Smirnov test, and when not normal, they were summarised by medians and interquartile ranges. “COPD” and “No COPD” groups were compared by Wilcoxon rank-sum test in quantitative variables and by chi squared pooled estimate of proportion test for categorical variables.

We evaluated the diagnostic accuracy of the spirometric variables using the receiver operating characteristic (ROC) curves. The threshold for each measure was determined based on the Youden index. We also reported the sensitivity and specificity obtained at the highest Youden index. A two-tailed p < 0.05 was considered statistically significant. Data were processed using MATLAB Release 2022a (The MathWorks, Inc., Natick, Massachusetts, United States).

### Ethical considerations

This study was conducted in compliance with the Declaration of Helsinki. It was approved by the Ethics Committee in Academic Research of the *Clinica Universidad de La Sabana,* in session #38, on the 4^th^ of May 2021. According to the applicable law (Resolution 8430 of 1993 for research in humans), the study was deemed to have no risk because it constitutes a documentary analysis, and no intervention on the patients was performed. Regarding the *Habeas Data* Law, Adriana Maldonado-Franco and Angélica Moreno-Giraldo were the only researchers with access permission to the full database with the patients’ full name and ID numbers.

## Results

The first round of the expert consensus process began with 48 variables classified into 26 questions. These 48 variables corresponded to information regarding the patients’ demographic characteristics, respiratory symptoms, and risk factors. After the first round, 19 variables were unanimously approved to be included in the COPD diagnostic process, while 17 were eliminated (supplementary table 1-3). The remaining 12 variables were included in the second round of the process, yielding two approved and two rejected. In the third round, experts analysed eight variables. The variables receiving the most expert acceptance votes were approved. Five more variables were included, while the rest were eliminated. Therefore, 26 clinical variables were incorporated into the web application for COPD diagnosis (table 1).

**Table 1:** Summary of demographic information, risk factors and respiratory symptoms that were included in the web application

No.	Variable
1	Age
2	Sex
3	Occupation
4	Smoker/Ex-smoker
5	Age when started smoking
6	Number of daily cigarettes
7	Age when stopped smoking
8	Packs per year
9	Passive smoker
10	Number of daily cigarettes of another smoker
11	Wood smoke exposure
12	Years of wood smoke exposure
13	Daily hours of wood smoke exposure
14	Previous diagnosis of COPD, chronic bronchitis, or emphysema
15	Previous diagnosis of asthma, asthmatic, or allergic bronchitis
16	Previous spirometry
17	Presence of respiratory symptoms
18	Age when respiratory symptoms began
19	History of atopy
20	Presence of wheezing
21	Frequency of wheezing
22	Presence of dyspnoea
23	Frequency of dyspnoea during physical activity
24	Presence of chronic cough
25	Chronic cough in the mornings
26	Chronic expectoration

COPD: Chronic Obstructive Pulmonary Disease

When designing the visualisation of the web application, we decided that the spirometric information should be presented in a format as similar as possible to the results delivered by the spirometer, so we included the trace labelled as the best during the test after applying the bronchodilator.

**Figure 1.**
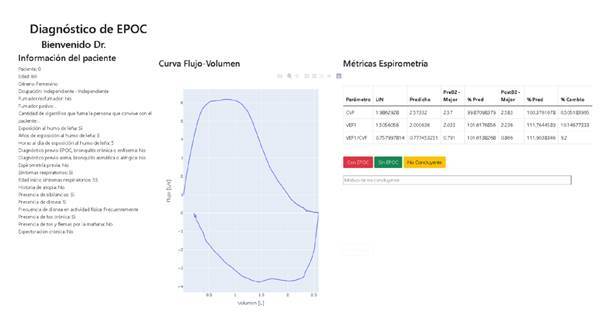
Screenshot of developed web application

We also included a table displaying the calculated lower limit of normal and predicted values for FVC, FEV_1_, and FEV_1_/FVC. The table also shows the best-measured values for these three metrics before and after applying the bronchodilator, and each value as a percentage of its corresponding predicted value. Finally, we added the percentage of change observed after applying the bronchodilator. Figure 1 shows a screenshot of the developed web application.

**Figure 2.**
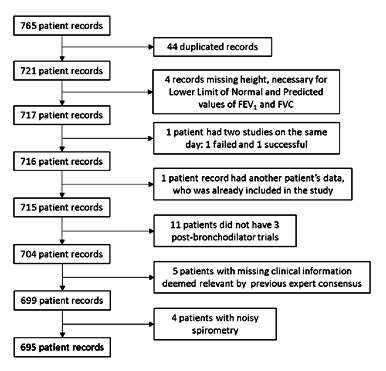
Patient selection process

FEV1: forced expiratory volume in the first second; FVC: forced vital capacity

The available database contained spirometric records and questionnaire responses from 765 patients. Upon examination, 44 duplicated records were identified and removed. Other excluded records were: one patient who underwent two separate spirometric tests on the same day, with only the second record included due to the first attempt failure; one patient record contained data from a different patient; four patients lacked height measurements; 11 patients had insufficient post-bronchodilator trials; five lacked relevant clinical data, and four had poor-quality spirometry. Following this selection process, 695 patient records remained for analysis (figure 2).

Out of the whole cohort, the expert classified all the patients with the information displayed in the web application: 34% (237/695) had COPD, 56% were women, and the median age was 66 years.

Most of the subjects (86%) reported having respiratory symptoms. Twentyseven percent reported having a previous COPD diagnosis, 14% stated they had been diagnosed with asthma, but less than half (46%) claimed they had gone through a spirometry study before (table 2).

**Table 2:** Baseline characteristics of the studied population

Variables	COPD	No COPD	
n^1^	237 (34)	458	(66)
Age^1^	71 (63-78)	63	(55-71)*
Women^2^	112 (47)	280	(61)*
Occupation - Homemaker^2^	88 (37)	175	(38)
Smoker/Ex-smoker^2^	124 (52)	192	(42)*
Years of smoking (without non-smokers)^1^	29 (13-41)	20	(10-33)*
Daily cigarettes (without non-smokers)^1^	9 (3-20)	4	(2-10)*
Packs per year (without non-smokers)^1^	10 (3-30)	4.98 (1.65-14.2)*	
Passive smoker^1^	42 (18)	100	(22)
Daily cigarettes of other smoker (only passive smokers)^1^	11 (4-20)	10	(5-20)
Wood smoke exposure^2^	160 (68)	249	(54)*
Years of wood smoke exposure^1^	20 (10-30)	17	(10-25)
Daily hours of wood smoke exposure^1^	5 (3-9.5)	6	(3-10)
Previous diagnosis of COPD, chronic bronchitis, or emphysema^2^	100 (42)	88	(19)*
Previous diagnosis of asthma, asthmatic, or allergic bronchitis^2^	38 (16)	58	(13)
Previous spirometry^2^	131 (55)	186	(41)*
Presence of respiratory symptoms^2^	209 (88)	386	(84)
Years with symptoms^1^	6 (2-20.75)	4	(2-12)*
History of atopy^2^	61 (26)	123	(27)
Presence of wheezing^2^	94 (40)	112	(24)*
Frequency of wheezing - sometimes^2^	72 (30)	96	(21)*
Presence of dyspnoea^2^	148 (62)	261	(57)
Frequency of dyspnoea during physical activity - very frequent^2^	65 (27)	89	(19)*
Presence of chronic cough^2^	116 (49)	204	(45)
Chronic cough in the morning^2^	92 (39)	126	(28)*
Chronic expectoration^2^	79 (33)	118	(26)*

COPD: Chronic Obstructive Pulmonary Disease

^1^ Median (Interquartile range)

^2^ N (%)

* p < 0.05

Wilcoxon rank sum test or chi square pooled estimate of proportion, as appropriate.

The COPD group had a median age of 71 years old, with a median of 6 years of having respiratory symptoms and dyspnoea as the most frequently reported symptom (62%). The most common risk factor was exposure to wood smoke (68%), followed by tobacco smoking (52%), with a median of packs per year of 29, without considering non-smokers (table 2).

## Discussion

Even though spirometry is a fundamental tool for diagnosing obstructive diseases, interpreting spirometry results alone is not enough to have a COPD diagnosis ^24^. Clinical information regarding the patient’s risk factors and symptoms are paramount to achieve an appropriate diagnosis. However, during the development of this study we have realised that primary care physicians have different needs when it comes to reaching a COPD diagnosis compared to pulmonologists. While specialists may be interested in a tool that helps them interpret the spirogram by itself, primary care clinicians might value a solution that not only considers the results of the spirometry but one that also helps them interpret risk factors and symptoms.

We added an option called “Non-conclusive” for those records without a clear diagnosis so the medical professional can input the reason for not reaching a decision. Thus, the patient can be derived for further analysis to confirm their diagnosis. In this study, the expert who tried the web application did not use this option but he suggested that it would be convenient to have the option to visualize all the available trial traces in the spirometric record before and after applying the bronchodilator. He found the application easy to use and suggested being able to adjust the axis of the spirometric graph and zoom in on areas of interest.

Among the strengths of our study, we found that the features of the population we studied represent those with the highest underdiagnosis and misdiagnosis rates in Latin America ^6^^,^^7^, making our study very relevant to our region. We also considered all included tests were performed in the same pulmonary laboratory, taken under the same conditions, with the same equipment, and by professionals in respiratory therapy who received the same training to reduce the amount of bias among studies.

It is important to mention that, for security reasons, this application requires the medical professional to log in with a username and password combination. The developer also has a username and password that allows access to download databases with the labels assigned by the medical professionals. These security measures guarantee database access only to those with permission. Also, during the diagnosis process, the expert was not able to see any patients’ identification, minimising the risk of bias and protecting patients’ privacy.

One of the limitations highlighted is that the study was conducted at a single research center. We found that the spirometer does not have a proper sample frequency according to the ATS/ERS standard ^25^. This feature may become problematic when analysing the signals using frequency-domain signal processing techniques such as the Fourier transform.

Also, the pulmonary laboratory where the testing was performed is not certified. We found that many records did not fulfill all the standard acceptance criteria, but they did comply those that make them usable ^25^. However, the information gathered through the various questionnaires enhanced the characterization of the data available on the web platform, enabling a broader and more detailed view of the evaluated patients ^7^^,^^13^^-^^20^. Regarding data selection by the expert consensus, it was based on a comprehensive evaluation of all available information, including data obtained from the questionnaires. This process influenced data interpretation and clinical decision-making.

In our research, only one expert determined the diagnosis based on the information in the web application. This fact could limit objectivity and interobserver variability in results interpretation, potentially affecting the diagnosis reliability. Additionally, the lack of multiple peer reviews could leave room for individual biases or interpretation errors. Finally, it would be interesting to carry out the expert consensus with a more significant sample of experts. Also, it would be good to test the accuracy of an expert using the web application in a cohort of patients with other diagnostic tests, such as computed tomography (CT) or diffusing capacity of the lungs for carbon monoxide (DLCO). However, the reasoning behind focusing on spirometry and clinical information is that COPD is a major burden in lower and middle-income countries, where spirometry is the only test available to diagnose the disease.

In the bibliographic research, we have found several computer-based solutions that use traditional statistics or more sophisticated techniques, such as machine-learning, to diagnose COPD using spirometry alone ^26^^-^^28^. We have also found some solutions aiming to define COPD severity ^29^ or predict COPD exacerbations ^30^ based on artificial intelligence techniques.

We have not found any other web-based solution contributing to COPD diagnosis by combining clinical history and spirometry. We hope that with all the information available in the web application we developed, a medical professional can have a full view of a patient’s condition, assisting their COPD diagnostic process. We believe this tool could become an important support resource in primary care and even allow remote consultation with experts. It could also be combined with machine-learning techniques, offering the probability of a COPD diagnosis based on the considered variables, improving timely diagnosis and management.

In conclusion, we developed a web application that displays demographic and clinical information as well as spirometric data to facilitate COPD diagnosis. Considering earlier disease manifestations might be evident in general practitioner offices, more technological tools must be developed and leveraged to increase timely COPD diagnosis rates.

## Archivos suplementarios

**Supplementary table 1.** Classification of variables in the first round of expert consensus

1 st round		
Accepted	Rejected	No decision
Age (years)	Race	Sex
Occupation	Weight (kg)	Height (cm)
Smoker/Ex-smoker	BMI (kg/m2)	Years of smoking
Age when started smoking	Weight, BMI (Categorical: < 25; 25.1-29.7; > 29.7]	Number of daily cigarettes of another smoker
Number of daily cigarettes	Education level	Daily hours of wood smoke exposure
Age when stopped smoking	Number of years in that level	Presence of wheezing
Packs per year (continuous variable)	Study years completed	Frequency of wheezing
Passive smoker	Last year in school	Wheezing in the last 12 months
Wood smoke exposure	Retired patient	Chronic expectoration
Years of wood smoke exposure	Smoked at least 100 cigarettes	Cough affected by weather
Previous diagnosis of COPD, chronic bronchitis, or emphysema	Packs per year (Categorical: 0-14; 15-24; 25-49; > 50)	Chronic cough in the morning
Previous diagnosis of asthma, asthmatic, or allergic bronchitis	Packs per year (Categorical: 0; < 20; 20-30; > 30)	Diminished life quality
Previous spirometry	History of allergies	
Presence of respiratory symptoms	Dyspnoea in the last four week	
Age when the respiratory symptoms began	Presence of phlegm or mucus	
History of atopy	Frequency of cough with phlegm	
Presence of dyspnoea	Cough and expectoration with cold weather	
Frequency of dyspnoea during physical activity		
Presence of chronic cough		

**Supplementary table 2.** Classification of variables in the second round of expert consensus

**2nd round**		
Accepted	Rejected	No decision
Daily hours of wood smoke exposure	Years of smoking	Sex
Chronic cough in the morning	Diminished life quality	Height (cm)
		Number of daily cigarettes of another smoker
		Presence of wheezing
		Frequency of wheezing
		Wheezing in the last 12 months
		Chronic expectoration
		Cough affected by weather

**Supplementary table 3.** Classification of variables in the third round of expert consensus

**3rd round**	
Accepted	Rejected
Sex	Height (cm)
Number of daily cigarettes of another smoker	Wheezing in the last 12 months
Presence of wheezing	Cough affected by weather
Frequency of wheezing	
Chronic expectoration	
